# Anisotropic Deformation Behavior and Indentation Size Effect of Monocrystalline BaF_2_ Using Nanoindentation

**DOI:** 10.3390/ma16196469

**Published:** 2023-09-29

**Authors:** Guangyuan Du, Xiaojing Yang, Jiayun Deng, Yanjun Guo, Tong Yao, Maozhong Li, Ruiwen Geng

**Affiliations:** 1 Faculty of Mechanical and Electrical Engineering, Kunming University of Science and Technology, Kunming 650500, China; kust_dgy@stu.kust.edu.cn (G.D.);; 2 Kunming Institute of Physics, Kunming 650233, China; 3 North Night Vision Science and Technology Research Institute Group Co., Ltd., Kunming 650217, China; 4 Hubei Engineering Research Center for Graphite Additive Manufacturing Technology and Equipment, China Three Gorges University, Yichang 443002, China

**Keywords:** nanoindentation, monocrystalline BaF_2_, anisotropy, deformation behavior, indentation size effect

## Abstract

In this study, our objective is to investigate the anisotropic deformation behavior and the indentation size effect (ISE) of monocrystalline barium fluoride (BaF_2_) using nanoindentation experiments with a diamond Berkovich indenter. BaF_2_ is known for its anisotropy, which results in significant variations in its mechanical properties. This anisotropy poses challenges in achieving high processing quality in ultra-precision machining. Through our experiments, we observed numerous pop-in events in the load–displacement curves, indicating the occurrence of plastic deformation in BaF_2_ crystals, specifically in the (100), (110), and (111) orientations; these pop-in events were observed as the indentation depth increased to 56.9 nm, 58.2 nm, and 57.8 nm, respectively. The hardness–displacement and elastic modulus–displacement curves were obtained from the tests exhibiting the ISE. The nanoindentation hardness of BaF_2_ is found to be highly dependent on its crystallographic orientation. Similarly, for BaF_2_ in the (100) orientation, the range is from 2.43 ± 0.74 and 1.24 ± 0.12 GPa. For BaF_2_ in the (110) orientation, the values range from 2.15 ± 0.66 to 1.18 ± 0.15 GPa. For BaF_2_ in the (111) orientation, the values range from 2.12 ± 0.53 GPa to 1.19 ± 0.12 GPa. These results highlight the significant influence of crystallographic orientation on the mechanical properties of BaF_2_. To better understand the ISE, we employed several models including Meyer’s law, the Nix–Gao model, the proportional specimen resistance (PSR) model, and the modified PSR (mPSR) model, and compared them with our experimental results. Among these models, the mPSR model demonstrated the best level of correlation ($R^{2}>0.9999$) with the experimental measurements, providing a reliable description of the ISE observed in BaF_2_. Our reports provide valuable insights into the anisotropic mechanical characteristics of BaF_2_ materials and serve as a theoretical guide for the ultra-precision machining of BaF_2_.

## 1. Introduction

Barium fluoride (BaF_2_) crystal is widely used in ultraviolet optical devices, infrared temperature measurement windows, CO_2_ laser windows, and the detection of high-energy photons (e.g., gamma rays and X-rays) due to its wide transmission range and scintillation properties among optically transparent ceramics [1,2,3]. Transparent monocrystalline BaF_2_ has mostly been considered for its higher light yield [4]. However, the manufacturing process for monocrystalline BaF_2_ poses challenges due to its soft-brittle nature and anisotropic effects. Grinding and polishing procedures often result in subsurface damage, thereby degrading the surface quality. To address this, it is crucial to employ a plastic regime removal mode during processing. Therefore, investigating the deformation behavior and the mechanical properties of monocrystalline BaF_2_ at the nanoscale becomes essential.

Nanoindentation technology has become a prominent method for studying the deformation behavior and mechanical properties of various materials at the nanoscale. This includes exploring the elastic–plastic transition [5,6,7], determining hardness and elastic modulus [8,9], and investigating the ISE where the hardness diminishes as the indentation depth increases [10,11,12,13]. In recent years, numerous studies have been conducted to investigate the deformation behavior and ISE of ceramics. Wang et al. [14] conducted nanoindentation experiments on sapphire crystals and developed a mechanical model to understand pop-in events. By utilizing critical stress values, they successfully predicted the critical load for pop-in events in spherical indenters with different tip radii. Importantly, their predicted results aligned well with experimental values, showcasing the reliability of their model for different crystal planes. Borc et al. [15] presented the characteristics of load–displacement curves and pop-in events on the (001) and (100) planes of potassium dihydrogen phosphate crystals under various loads. Their findings revealed that the (100) plane exhibited fewer pop-in events compared to the (001) plane, with the indentation displacement values ranging from 3 to 20 nm. In another study, Zhu et al. [16] developed a molecular dynamics simulation model to investigate the deformation behavior of 4H silicon carbide (4H-SiC) during nanoindentation. They observed the appearance of pop-in events in the load–displacement curves and found that the vertical deformation pattern during 4H-SiC indentation manifested as small pop-in events in the P-h curves. Yan et al. [17] focused on the ISE and examined the hardness and elastic modulus of the sapphire (10-10) plane. They employed the Nix–Gao (N-G) model and the proportional specimen resistance (PSR) model. Upon indenting the monocrystalline sapphire (10-10) plane, they observed a substantial ISE/non-ISE region and obtained real hardness values from different deformation regions. Using nanoindentation measurements, Petrus et al. [18] investigated the ISE of high-entropy carbides. using nanoindentation measurements. They analyzed the relationship between hardness and load by applying the conventional Meyer’s law (ML), PSR model, and modified proportional specimen resistance (mPSR) model. Their study concluded that the material’s true hardness and ISE played significant roles in plastic deformation and the development of microcracks during nanoindentation. Maiti et al. [19] explored the ISE in zirconia-toughened alumina ceramics through nanoindentation experiments. They critically evaluated the ISE using the elastic recovery model, PSR model, mPSR model, and N-G model. Their analysis led to the conclusion that the N-G model provided a better explanation for the observed ISE phenomena.

However, the deformation behavior and mechanical properties of monocrystalline BaF_2_ are still not well understood. Therefore, this study aims to investigate these aspects by conducting nanoindentation experiments and developing theoretical models. Specifically, we focus on the (100), (110), and (111) planes of BaF_2_ monocrystals. Our objective is to analyze the occurrence of pop-in events, the elastic–plastic transition, and explore the ISE. To begin, we analyze the anisotropic deformation process during the initial stage of indentation. We quantify the first pop-in event and confirm that the deformation initiation stage is purely elastic. Subsequently, we accurately estimate the indentation hardness and elastic modulus of monocrystalline BaF_2_. Furthermore, we delve into the ISE by employing several models, namely ML, the N-G model, the PSR model, and the mPSR model. Through our investigation, we uncover a pronounced anisotropic ISE that manifests during nanoindentation. The primary aim of this study is to deepen our understanding of the anisotropic deformation behavior and ISE exhibited by monocrystalline BaF_2_.

## 2. Experimental Procedures

**Figure 1:**
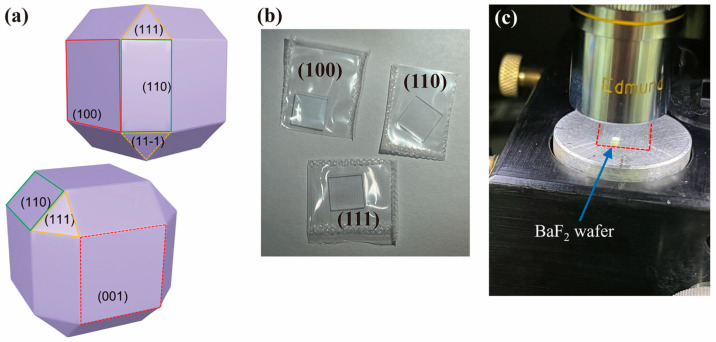
Experimental BaF_2_ wafers. (**a**) Prominent planes in FCC structure of monocrystalline BaF_2_. (**b**) (100), (110), (111) crystal plane-orientated and polished single-crystal BaF_2_ wafers. (**c**) Material waxed onto metal block, and mounted on Nano Indenter G200.

The BaF_2_ wafers were supplied by HF-Kejing Materials Technology Co., Ltd. (Hefei, China) Three different sample types, including (100), (110), and (111) with various crystallographic planes (Figure 1a), were meticulously polished, and their surface roughness is not more than 10 Å. The size of each crystal plane is 10 mm × 10 mm × 0.5 mm (Figure 1b). An X-ray photoemission spectroscopy analysis report of the samples provided by HF-Kejing showed that no other elements were present in the materials.

**Figure 2:**
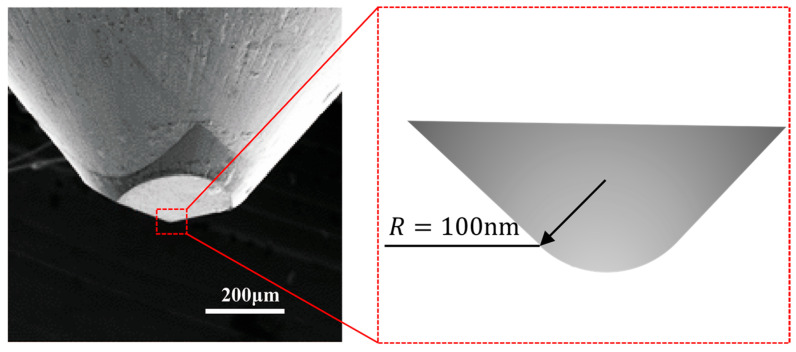
SEM of the Berkovich tip and its radius R=100 nm.

To ensure the reliability of the indentation results, the wafers were marked with an indentation area using a light microscope. Then, the marked wafers were waxed onto a metal block to start the indentation experiment (Figure 1c). The Berkovich tip is part of the indentation testing equipment (Nano Indenter G200, Agilent/Keysight Technologies, Inc., Santa Clara, CA, USA), with the tip radius R being 100 nm (Figure 2).

Constant strain rate testing was employed in the nano-indentation experiment to obtain the mechanical properties of each BaF_2_ plane. Sets of indentation tests were carried out on the (100), (110), and (111) crystal planes, with a fixed strain rate of $\varepsilon =0.05\;s^{-1}$. The maximum displacements for the 7 series were 50 nm, 100 nm, 200 nm, 500 nm, 1000 nm, 2000 nm, and 4000 nm, respectively. The loading rate ε is defined as [20]:

(1) $$\varepsilon =\frac{\dot{P}}{P}$$

where P is the load on the sample. The load is held for 10 s before the unloading process. 

An essential part of the experiments in this study is continuous stiffness measurement [21], which is used to quantify the contact stiffness S during loading to exclude the impacts of thermal drift on the lower strain rate data. S is defined as the differentiation of the unloading curve fit, which is given by Equation (2).

(2) $$S={\left(\frac{dP}{dh}\right)}_{h=h_{max}}$$

Five tests were conducted at each indentation depth in accordance with ISO 14,577 [22], to collect repeatable and reproducible results. To reduce the raw error caused by thermal drift, the drift correction was kept below 0.5 nm/s. 

**Figure 3:**
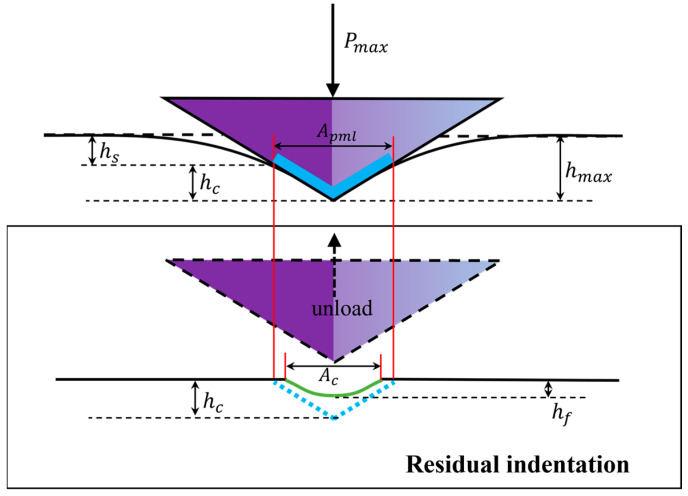
Schematic diagram of mechanical parameters of nanoindentation.

A schematic diagram of the mechanical parameters is presented in Figure 3, in which h is the design indentation depth, $P_{max}$ is the peak load, and $h_{c}$ is the contact depth.

They are estimated according to

(3) $$h_{c}=h_{max}-h_{s}=h_{max}-a\frac{P_{max}}{S}$$

Here, a is a constant that depends on the shape of the tip. For a Berkovich tip, a=0.75; $h_{f}$ is the residual penetration depth.

Oliver and Pharr introduced a method in the 1990s to precisely determine H and E from the indentation load–displacement data from the indentation without requiring measurement of the deformed region under a microscope [21]. In this study, the hardness H and elastic modulus E are determined using the Oliver–Pharr model. In nanoindentation, the hardness H of the material is defined as $H=P_{max}/A_{pml}$, where $A_{pml}$ is the residual contact area and is determined by the area function $A=f(h_{c})$. This function is sometimes referred to as the indenter shape function. For an ideal Berkovich indenter:

(4) $$\;A_{pml}=3\sqrt{3}\tan^{2}\frac{\alpha }{2}h_{c}^{2}=24.56h_{c}^{2}$$

where the apex angle (α) of the Berkovich indenter is 130.6°.

Due to limitations in machining and grinding technology, as well as wear during use, the tip of the indenter often deviates from the ideal situation. In fact, the indenter tip cannot be considered a geometric point in the strict sense but can be approximated as a *small* spherical surface. Therefore, it is necessary to modify the actual tip’s area function based on the ideal surface basis function. A general polynomial form is used for this modification:

(5) $$A_{pml}=f\left(h_{c}\right)=24.56h_{c}^{2}+C_{1}h_{c}^{1}+C_{2}h_{c}^{\frac{1}{2}}+C_{3}h_{c}^{\frac{1}{4}}+\cdots$$

The fitting parameters $C_{i}$ can be determined by performing nanoindentation tests on samples. The contact’s reduced elastic modulus $E_{r}$ is represented by

(6) $$E_{r}=\frac{\sqrt{\pi }}{2\beta }\frac{S}{\sqrt{A}}$$

Here, β is a constant determined by the profile of the tip. In this experiment, β=1.034 for the Berkovich tip. The elastic modulus is obtained using the following equation:

(7) $$\frac{1}{E_{r}}=\frac{1-v^{2}}{E}+\frac{1-v_{i}^{2}}{E_{i}}$$

In Equation (7), v and E represent the Poisson’s ratio and elastic modulus of the sample, respectively. $v_{i}$ and $E_{i}$ represent the Poisson’s ratio and elastic modulus of the indenter, respectively. In this experiment, the Poisson’s ratio of single crystal BaF_2_ is 0.343. For the diamond Berkovich tip, the commonly used values are $E_{i}=1141\;Gpa$ and $v_{i}=0.07$. The data obtained from this experiment are based on the above theoretical equations and relevant parameters.

## 3. Results and Discussion

The results and analyses of the three planes of monocrystalline BaF_2_ are presented in a systematic manner. Firstly, descriptions of the indentation topography and residual indentation are provided, as they play a crucial role in the subsequent application and interpretation of the model. Secondly, instrumental indentation results for the elastic modulus and hardness are presented and represented as characteristic curves. Thirdly, a quantitative theoretical analysis is conducted to examine the microscopic phenomena observed in the aforementioned curves. Finally, the size effects observed during indentation are thoroughly analyzed and validated using different models.

### 3.1. Indentation Morphology

**Figure 4:**
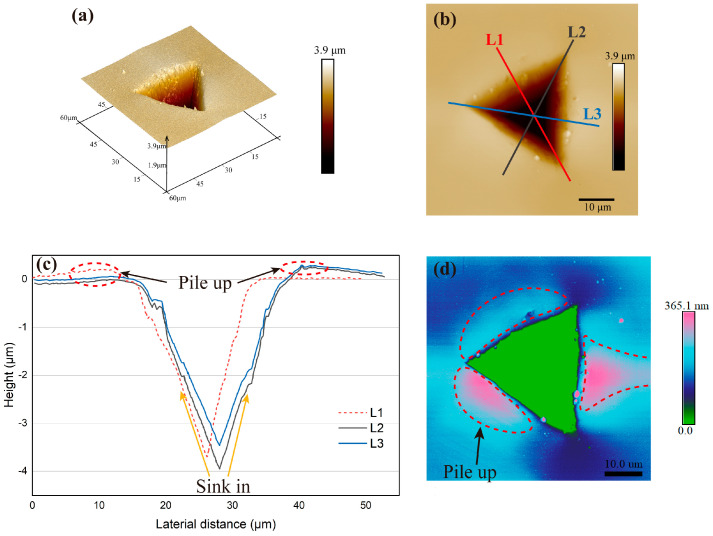
AFM images of (**a**) 3D view of BaF_2_ (111) indentation profile with maximum indentation depth of 4000 nm, (**b**) schematic diagram of the relative positions of the three cross-sections, (**c**) line profile (three different sections) obtained after indentation experiment at $P_{max}=366.61,$ and (**d**) pile-ups at the edge of the nanoindentation crater.

The topography of the Berkovich indent surface on the (111) plane of BaF_2_ monocrystalline, as well as the indent morphology at an indentation depth of approximately 4000 nm, are precisely described using Atomic Force Microscopy (AFM) (Figure 4).

At the aforementioned h, the residual depth and pile-up height were measured to be 3928.0 nm and 365.1 nm, respectively (Figure 4c,d). The presence of concave edges at the contact peripheries in Figure 4c,d suggests material pile-up. The AFM data in Figure 4c reveal the sink-in of material at the indenter faces. The occurrence of pile-up can be attributed to the conventional shear-induced flow of material. Furthermore, the analysis of the AFM images and software data processing (Figure 4b) confirms that no cracks were generated at the edge of the nanoindentation crater. 

### 3.2. Indentation Load vs. Displacement Curves and Plastic Deformation

**Figure 5:**
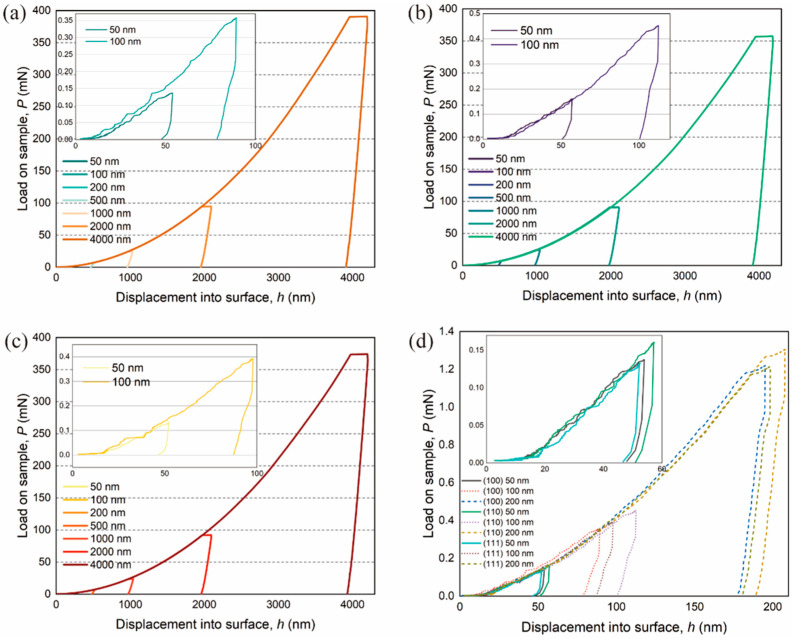
Nanoindentation P vs. h curves of BaF_2_ (100), (110), and (111) with Berkovich indenter in CSR-mode: (**a**) BaF_2_ (100); (**b**) BaF_2_ (110); (**c**) BaF_2_ (111); (**d**) P vs. h curves of BaF_2_ at 50 to 200 nm on three crystal planes are zoomed in.

Moving on to the indentation load, P vs. indentation depth h for BaF_2_ (100), (110), and (111) obtained by nanoindentation to different maximum indentation depths $h_{max}$ are illustrated in Figure 5.

It is demonstrated that the maximum load of BaF_2_ differs for each indentation depth and crystal plane, and the unloading curves do not return to their original positions. When the indentation depth is below 100 nm, the P vs. h curves for each crystal plane are very close (Figure 5d). However, as the indentation depth exceeds 100 nm, particularly at 4000 nm, distinct characteristics in the P vs. h curves emerge for the three crystal planes. This observation aligns with nanoindentation simulations and experiments conducted on other single-crystal materials [15,23,24]. 

#### 3.2.1. Pop-In Events

**Figure 6:**
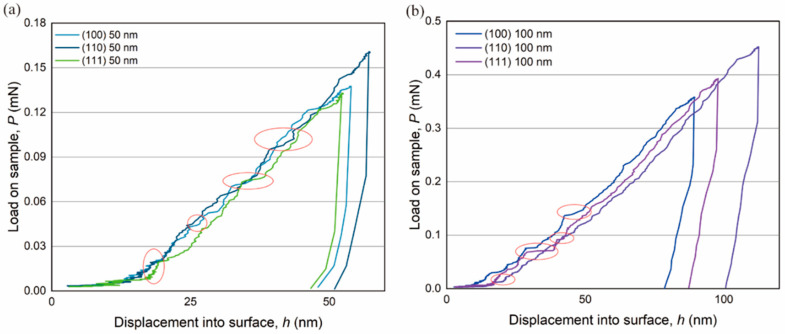
Load on sample, P vs. indentation depth, h curves of BaF_2_ (100), BaF_2_ (110), and BaF_2_ (111) at maximum designed depth of (**a**) 50 nm and (**b**) 100 nm shown.

Figure 6 shows that distinct crystal planes experience several pop-in events (red circles) during the loading process.

Representative P vs. h curves for all three crystal planes are arranged together in Figure 6 to compare the consecutive pop-in behaviors of the various crystal planes. All the planes were subjected to several peak loads ($P_{max}$) ranging from 0.133 to 0.160 mN (Figure 6a). According to nanoindentation-based nanomechanical characterization, pop-in bursts occur between 0.02 and 0.12 mN at different $P_{max}$. The onset loads for the first pop-in are 11.0 μN, 19.0 μN, and 7.0 μN at the penetration depths of 14 nm, 18 nm, and 10 nm for the BaF_2_ (100), (110), and (111) planes. This indicates that the nanoindentation is in a purely elastic deformation stage at this point. After the first pop-in event, we also observed successive pop-in events on the P vs. h curves in our test, indicating the transition from purely elastic deformation to elastic–plastic deformation stage.

#### 3.2.2. Elastic–Plastic Transmission

Regarding the elastic–plastic transition, we use the Hertz analysis [25] to explain it. In this theoretical model, we assume two contacting objects with radii $R_{1}$ and $R_{2}$, and the contact radius a can be estimated as

(8) $$a=\frac{\pi p_{0}R_{0}}{2E_{r}}$$

with the effective curvature $R_{0}$.

(9) $$\frac{1}{R_{0}}=\frac{1}{R_{1}}+\frac{1}{R_{2}}$$

Here, $p_{0}$ is the maximum contact pressure and is specified as [26] $p_{0}=3/2\;p_{m}$, where $p_{m}$ is the mean contact pressure. The value of $p_{0}$ for yield is supplied by Tresca’s criterion:

(10) $${\left(p_{0}\right)}_{yield}=\frac{3}{2}{\left(p_{m}\right)}_{yield}=1.6Y$$

where Y is the yield stress ($Y=\frac{H\left(Hardness\right)}{2.8}$). The critical depth ($\delta_{yield}$) of the elastic–plastic transition in the nanoindentation is 0.48a [26]. Combining Equations (8)–(10), we can express $\delta_{yield}$ as

(11) $$\delta_{yield}=0.48\frac{\pi {\left(p_{0}\right)}_{yield}R_{0}}{2E_{r}}=0.1371\frac{\pi HR_{0}}{E_{r}}$$

In this study, we calculate $\delta_{yield}$ for the monocrystalline BaF_2_ (100), (110), and (111) planes as 56.9 nm, 58.2 nm, and 57.8 nm, respectively. The comparison between *a* and *b* in Figure 5 shows that the measured starting load at the appearance of the first pop-in event is almost independent of $P_{max}$. Previous studies [7,8] on pop-in events have illustrated that dislocation nucleation and sliding are inherent characteristics of FCC crystals. Therefore, the chosen $P_{max}$ has no bearing on the subsequent pop-in events, as long as the maximum load is higher than the actual load of the pop-in events.

### 3.3. Anisotropy ISE of Monocrystalline BaF_2_

#### 3.3.1. Mechanical Properties

**Figure 7:**
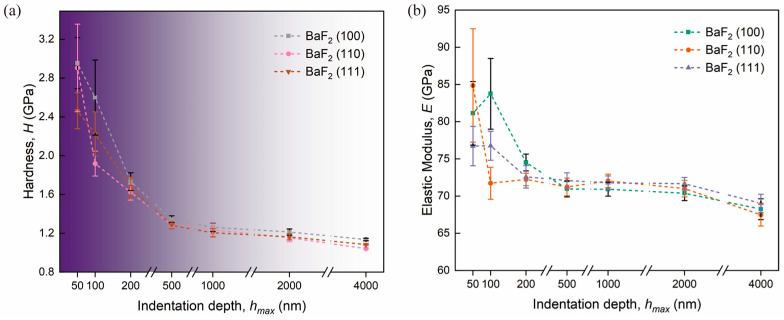
Variation of nanoindentation hardness and elastic modulus ((**a**,**b**), respectively) with respect to the maximum indentation depth ($h_{max}$) for monocrystalline BaF_2_ (100), (110), and (111).

The plot in Figure 7 demonstrates the variation of nanoindentation hardness (H) and elastic modulus (E) with maximum indentation depth ($h_{max}$).

The elastic modulus values for BaF_2_ (100), (110), and (111) planes were measured as 81.12 ± 4.27 GPa, 85.02 ± 7.62 GPa, and 76.72 ± 2.65 GPa, respectively, within the fully elastic region (≤60 nm) (Figure 7b). However, as the indentation depth increased, the indentation modulus (E) values appeared to remain relatively constant. This behavior is consistent with what is observed in other crystalline materials [8,27,28,29,30]. No significant change in elastic modulus was observed after pop-ins, with average values of E for BaF_2_ (100), (110), and (111) being approximately 69.85 ± 3.31 GPa, 70.17 ± 3.54 GPa, and 70.82 ± 3.02 GPa, respectively. The overestimation of E can be attributed to the roundness of the Berkovich indenter tip [31]. 

In Figure 7a, it can be observed that the hardness (H) decreases with increasing maximum indentation depth ($h_{max}$) for all planes, indicating the presence of the ISE. Additionally, BaF_2_ (100), (110), and (111) exhibited higher Oliver–Pharr hardness ($H_{OP}$) values in the purely elastic region, namely 2.95 ± 0.26 GPa, 2.90 ± 0.45 GPa, and 2.46 ± 0.19 GPa, respectively (Figure 7a). However, the elastic–plastic contact occurred at depths between 10 and 60 nm, whereas the ISE ranges from approximately 10 to 200 nm. The depth dependency of $H_{OP}$ becomes irrelevant as the indentation depth exceeds 200 nm, entering a non-ISE region where the hardness remains constant there. For BaF_2_ (100), (110), and (111), the mean $H_{OP}$ values in the non-ISE and ISE regions were calculated as 2.43 ± 0.74 and 1.24 ± 0.12 GPa, 2.15 ± 0.66 and 1.18 ± 0.15 GPa, 2.12 ± 0.53 GPa and 1.19 ± 0.12 GPa, respectively. Where the hardness and elastic modulus of the BaF_2_ (100) in the non-ISE region are comparable to the previous study of Morris et al. [32] and Gill et al. [33] During the early stage of indentation, the indenter tip applies force solely to the material’s upper surface, where the hardness and elastic modulus predominantly depend on the stress distribution within the surface layer. Consequently, there is a significant decrease in the nanohardness and elastic modulus in the low-load region [34]. As the indentation depth increases, the work performed by the indenter transforms [35,36].

To evaluate the effectiveness of existing theories in explaining the anisotropic ISE observed in monocrystalline BaF_2_, we considered Meyer’s law, the Nix–Gao model (plastic gradient theory), the proportional specimen resistance (PSR) model, and the modified PSR model. The results of the efforts are listed below in chronological order.

#### 3.3.2. Meyer’s Law (ML)

**Figure 8:**
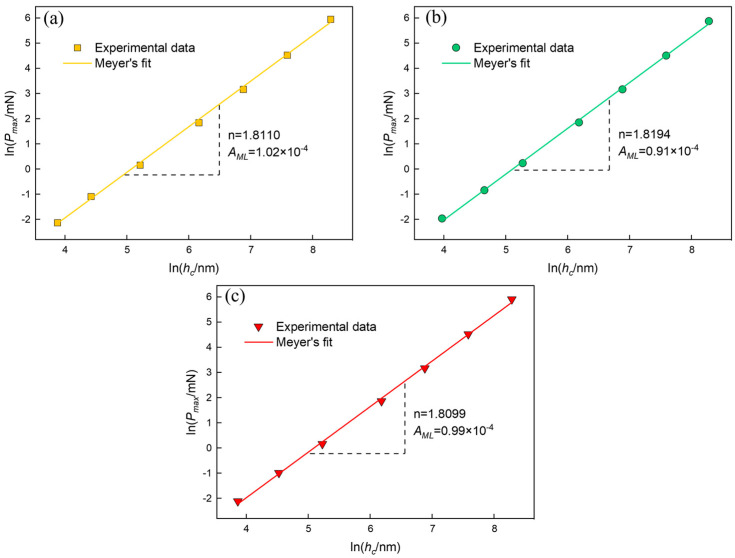
The log of maximum indentation load ($\ln P_{max}$) is plotted against the log of contact depth ($\ln h_{c}$), which is based on Meyer’s law: (**a**) BaF_2_ (100); (**b**) BaF_2_ (110); (**c**) BaF_2_ (111).

Meyer’s law is the simplest way to describe the ISE. The relation between the maximum indentation load ($P_{max}$) and contact depth ($h_{c}$) is given as follows [37]:

(12) $$P_{max}=A_{ML}h_{c}^{n}$$

where $A_{ML}$ is a subjective constant and n is the hardening coefficient. The Meyer’s graph (Figure 8) of $\ln P_{max}$ vs. $\ln h_{c}$ provides the value of n, and least squares fitting of the data yields straight line graphs with a high correlation coefficient ($R^{2}\sim 0.9999$).

As illustrated in Figure 8, the exponent n for BaF_2_ (100), (110), and (111) is 1.8110, 1.8194, and 1.8099, respectively. An exponent n<2 is appropriate for typical ISE behavior.

When n>2, there is a reverse ISE behavior, and when n equals to 2, the hardness is independent of the applied stress, indicating no ISE. This observation is consistent with the H value reported in Figure 7a. For monocrystalline ceramics, lower n values are typically correlated with higher $A_{ML}$ values [38,39] and the hardness of the material. The analysis leads to the conclusion that the ISE of BaF_2_ wafers at various crystallographic planes is well described by Meyer’s law. One of the reasons for this is that the experiments used a lower indentation load, preventing crack development during the indentation process. Although Meyer’s law can partly reflect the ISE of monocrystalline BaF_2_, the physical significance of the parameters $A_{ML}$ and n in this model is not accurate, and the true hardness of monocrystalline BaF_2_ cannot be calculated without considering the ISE. Hence, further investigation is required to understand the relationship between P and h. 

#### 3.3.3. Nix–Gao (N-G) Model

**Figure 9:**
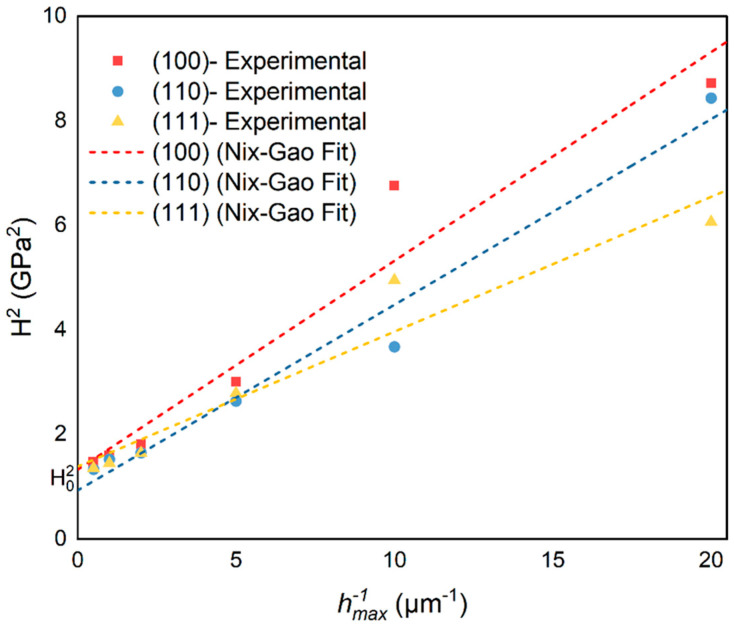
Plots of the BaF_2_ (100), (110), and (111) data obtained experimentally. The reciprocal of the maximum indentation depth ($h_{max}^{-1}$) and the square of indentation hardness ($H^{2}$) are fitted.

The N-G model, which is based on the plastic gradient theory, has been widely utilized to explain the relationship between ISE and hardness values. According to the plastic gradient plasticity model proposed by Nix and Gao [40], when the material is subjected to indenter loading, the resulting indentation arises from the formation of a dislocation loop. This loop is generated by statistically stored dislocations and geometrically necessary dislocations (GNDs) within the material beneath the indenter. As a result, the strain gradient effect causes changes in the distribution of GNDs during the loading process, leading to the appearance of the ISE. Small indentations have high strain gradients that result in GNDs that increase hardness due to the presence of GNDs [40]. The relationship between hardness (H) and the maximum indentation depth ($h_{max}$) can be expressed as follows [40]:

(13) $$\frac{H^{2}}{H_{0}^{2}}=1+\frac{h^{*}}{h_{max}}$$

where H is the material’s hardness as measured by the experiment; $H_{0}$ is its hardness without taking account of the GNDs and $h^{*}$ is the length scale corresponding to the material’s characteristics:

(14) $$h^{*}=\frac{81}{2}b\alpha^{2}\tan^{2}\left(90-\theta \right){\left(\frac{\mu }{H_{0}}\right)}^{2}$$

where b, α, θ, and μ refer to the Burger vector of GNDs, dislocation–dislocation interaction parameter, semi-apex angle of indenter, and shear modulus of the material being indented, respectively. The plot of $H^{2}$ vs. $h_{max}^{-1}$ (Figure 9) yielded the constants $H_{0}$ and $h^{*}$ of Equation (8), which are reported in Table 1.

The macroscopic hardness squared $H_{0}^{2}$ is what the y-axis intercept on the graph represents. The regression coefficient values in this table (Table 1) show the data’s departure from the N-G fit.

Evidently, when h is less than $h^{*}$, a significant ISE phenomenon is observed in monocrystalline BaF_2_. When h exceeds $h^{*}$, the ISE of monocrystalline BaF_2_ diminishes. Values of $H_{0}$ and $h^{*}$ evaluated using the N-G model are higher and very close to the experimental results (Figure 7a) because this model is based on the assumption of ideal materials. 

#### 3.3.4. Proportional Specimen Resistance (PSR) Model

**Figure 10:**
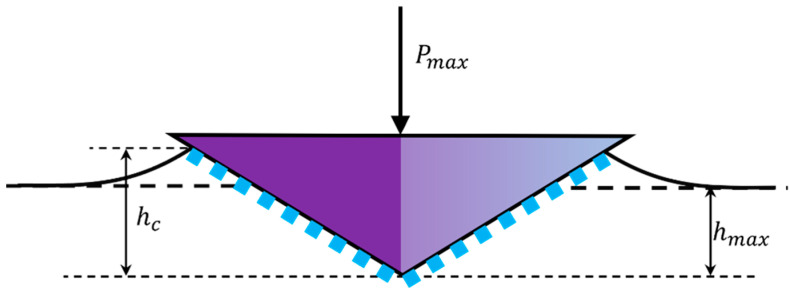
Schematic diagram of the indentation where plastic pile-up exists.

It has been argued by some researchers that the discrepancy between the actual contact area ($A_{c}$) of the indentation and the projected area ($A_{p}$) is of a geometric difference. However, this geometric approximation holds true only in cases where the indentation leads to complete plastic deformation [41]. It is important to note that Equations (3) and (4) do not consider the plastic phenomenon of pile-up (Figure 10), as they are based on the assumption that the contact depth is smaller than the indentation depth.

In this study, monocrystalline BaF_2_ exhibits plastic deformation and pile-up (Figure 4c,d) at indentation depths exceeding 100 nm. Therefore, the proportional specimen resistance (PSR) model is used to further describe the elastic–plastic properties [42]. The PSR model is applied to mathematically calculate the true hardness and is represented as [17,38]:

(15) $$\frac{P_{max}}{h_{c}}=a_{1}+a_{2}h_{c}$$

**Figure 11:**
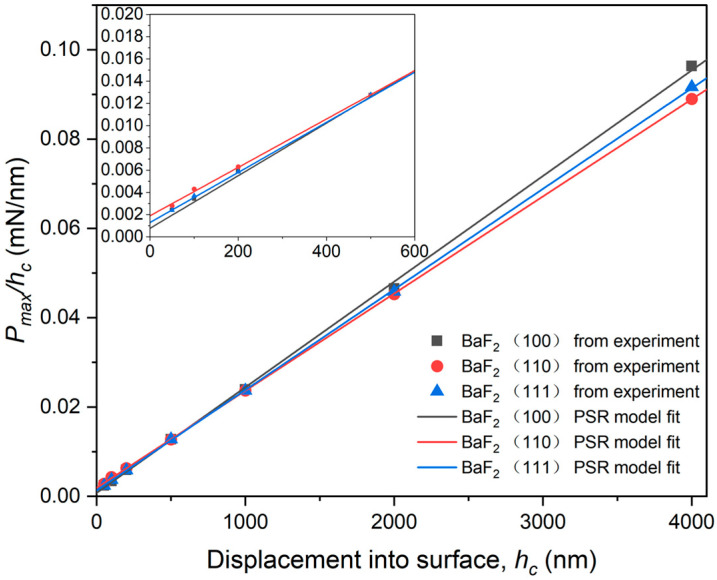
Fitting curves of $P_{max}/h_{c}$ against $h_{c}$ based on PSR model.

The variables $a_{1}$ and $a_{2}$ are connected to the elastic and plastic properties of the materials, as mentioned in Equation (15). These variables represent the intercept and slope, respectively, of the plot between $P_{max}/h_{c}$ and $h_{c}$ illustrated in Figure 11.

They are recommended as measures of the true hardness, denoted as *H*_0_. To assess the feasibility of the PSR model in evaluating the ISE, two sets of $H_{0}$ values were obtained for BaF_2_ (100), (110), and (111) [17]:

(16) $${\left(H_{0}\right)}_{1}=\frac{P_{max}-a_{1}h_{c}}{24.5h_{c}^{2}}$$

(17) $${\left(H_{0}\right)}_{2}=\frac{a_{2}}{24.5}$$

were produced for BaF_2_ (100), (110), and (111). The results obtained from the aforementioned Equation (17) to Equation (15) are listed in Table 1. Both sets of true hardness values, denoted as ${\left(H_{0}\right)}_{1}$ and ${\left(H_{0}\right)}_{2}$, exhibit close proximity to each other. For BaF_2_ (100), (110), and (111), the values of ${\left(H_{0}\right)}_{1}$ are 0.899 ± 0.29 GPa, 0.816 ± 0.31 GPa, and 0.853 ± 0.25 GPa, while the values of ${\left(H_{0}\right)}_{2}$ are 0.967 GPa, 0.890 GPa, and 0.918 GPa, respectively (with the correlation coefficient close to 1, as listed in Table 2). These results indicate a strong agreement between the predictions of the model and the experimental findings. Consequently, within the specified load range, the PSR model effectively describes the ISE observed in monocrystalline BaF_2_. 

Analytically, the $H_{OP}$ values in the fully elastic deformed region range from 2.46 to 2.95 GPa. Furthermore, in the ISE region (h≤200 nm), $H_{OP}$ is heavily dependent on the indentation depth. Additionally, the N-G model demonstrates the existence of ISE at the critical pop-in loads. When the applied load exceeds the critical pop-in load, the N-G model becomes applicable, resulting in the development of a plastic zone. However, experimental tests reveal that the actual plastic zone $A_{c}$ is smaller than what is predicted by the N-G model. As a result, the N-G model overestimates $H_{OP}$ at substantially lower depths. Higher $H_{OP}$ values may also contribute to the formation of pile-ups at relatively shallow indentation depths. In comparison, the PSR model, which provides depth-independent hardness values, demonstrates a strong correlation coefficient ($R^{2}>0.99$). This is noteworthy even though the $H_{OP}$ values calculated by the N-G model also show good agreement. 

#### 3.3.5. The Modified PSR Model

Residual stresses are often present on the surface of polished monocrystalline BaF_2_ wafers. These residual stresses interact with the surface roughness, which can impact the measured values of nanoindentation hardness obtained from experiments. Unfortunately, the conventional PSR model fails to account for this significant effect. To address this limitation, we propose the use of the modified PSR (mPSR) model, which considers the interaction of residual stresses with the surface toughness in all cases. The mPSR model introduces a corrective parameter to account for these interactions, providing a more accurate description of the ISE. The mathematical expression of the mPSR model is given by the following equation [38,43]:

(18) $$P_{max}=a_{0}+a_{1}h_{c}+a_{2}h_{c}^{2}$$

**Figure 12:**
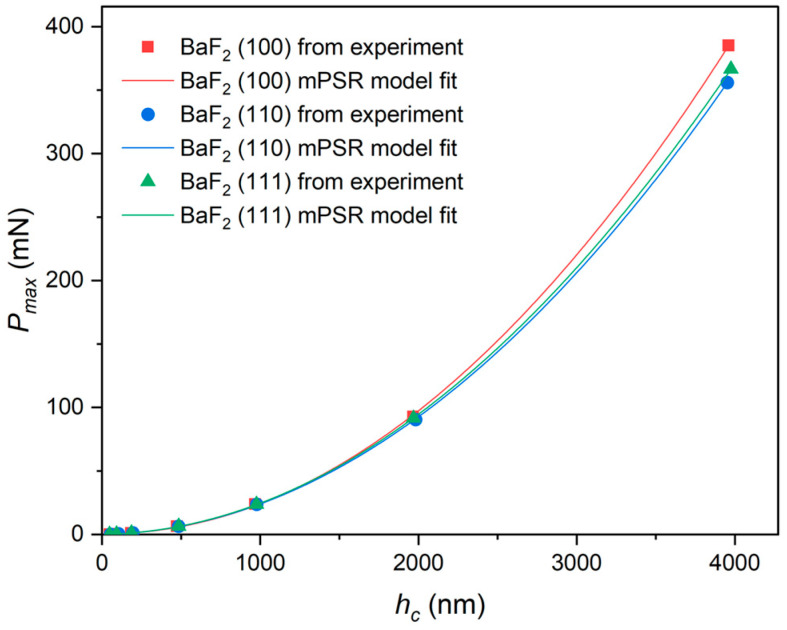
Based on the mPSR model, fitting curves of the experimental data measured $P_{max}\;vs.\;h_{c}$ of monocrystalline BaF_2_ to Equations (18)–(20) give best-fit outcomes after fitting polynomial regression models traditionally.

In Equation (18), $a_{1}$ and $a_{2}$ refer to the same meaning as in Equation (15). $a_{0}$ is an additional parameter introduced, which, unlike $a_{1}$ and $a_{2}$, is related to the residual stresses generated on the material surface during the polishing process. The parameters $a_{0}$, $a_{1}$, and $a_{2}$ are obtained by fitting a polynomial curve to the relationship between $P_{max}$ and $h_{c}$, as illustrated in Figure 12.

The obtained data are summarized in Table 3.

The true hardness values (${\left(H_{0}\right)}_{1}$ and ${\left(H_{0}\right)}_{2}$) are similar to those predicted by the PSR model and are given by the following equations:

(19) $${\left(H_{0}\right)}_{1}=\frac{P_{max}-a_{0}-a_{1}h_{c}}{24.5h_{c}^{2}}$$

(20) $${\left(H_{0}\right)}_{2}=\frac{a_{2}}{24.5}$$

From the mPSR model, the values of ${\left(H_{0}\right)}_{1}$ and ${\left(H_{0}\right)}_{2}$ for BaF_2_ (100), (110), and (111) are approximately 1.01 ± 0.098 GPa, 0.915 ± 0.092 GPa, 0.928 ± 0.094 GPa, 1.012 GPa, 0.914 GPa, and 0.927 GPa for BaF_2_ (100), (110), and (111), respectively (with the correlation coefficient close to 1, as listed in Table 3). It is worth noting that the parameter values of $a_{0}$ related to residual stress are positive for each plane of monocrystalline BaF_2_, indicating a tendency for compression on all three surfaces. However, the parameter value of $a_{1}$ is negative. The cause of this is unknown, and further focused effort beyond the scope of the current work should be undertaken to investigate it. By combining the N-G model, the PSR model, and the mPSR model, all three models effectively explain the ISE to some extent and the predicted true indentation hardness values are lesser than the experimentally obtained indentation hardness ($H_{OP}$) values.

#### 3.3.6. Quantification of Size Effects

To investigate the quantification of the size effect in monocrystalline, Manika [44] confirmed the power law–exponent relationship (at a low-load range) between nanohardness (H) and indentation depth (h) which can be summarized as follows:

(21) $$H=C\cdot h^{-m}$$

where C is a constant that only affects the indenter’s shape factor, m is the power law–exponent or the ISE index. h is the depth of the indentation.

**Figure 13:**
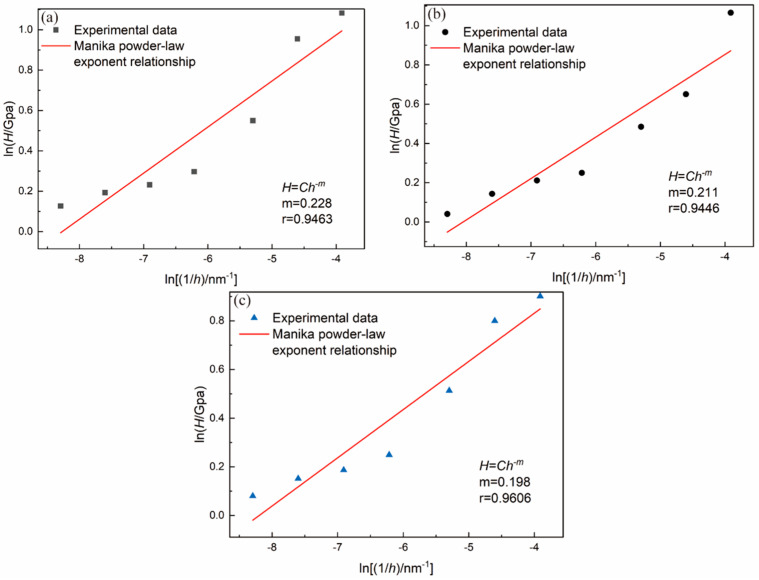
Fittings of the relationship between H and 1/h and the experimentally obtained nanoindentation data: (**a**) BaF_2_ (100); (**b**) BaF_2_ (110); (**c**) BaF_2_ (111).

By using the relationship described in Equation (21) and performing a linear regression analysis between ln(*H*) and ln(1/*h*), the value of *m* can be determined. The fitted curves connecting H to 1/h are illustrated in Figure 13.

Based on the results of the fitting, the values of m for BaF_2_ (100), (110), and (111) were found to be 0.228, 0.211, and 0.198, respectively. It is worth noting that in the case of metal and semiconductor materials, the value of m typically falls within the range from 0.12 to 0.32. Additionally, a smaller value of m tends to indicate improved plastic properties of the material [44]. Based on this study, BaF_2_ (100) exhibits the highest size effect index m and hardness among BaF_2_ (100) and BaF_2_ (111). Therefore, it can be inferred that BaF_2_ (100) has relatively poorer plasticity. 

## 4. Conclusions

Nanoindentation experiments were conducted on monocrystalline BaF_2_ using a Berkovich diamond indenter. The aim was to study the anisotropic deformation behavior and ISE of BaF_2_ during the nanoindentation. The conclusions drawn from the study are as follows:

1. Successive pop-in events were observed on the load-depth curves, and a pile-up phenomenon was observed based on AFM images. The onset loads for the first pop-in were found to be 11.0 μN, 19.0 μN, and 7.0 μN for the (100), (110), and (111) crystallographic planes, respectively. Among these planes, the (100) plane required BaF_2_ (110) and BaF_2_ (111), BaF_2_ (100) necessitates the highest load at a given indentation depth. The critical depth of elastic-plastic deformation for monocrystalline BaF_2_ was determined to be 56.9 nm, 58.2 nm, and 57.8 nm for the (100), (110), and (111) planes, respectively.
2. Each crystallographic plane of monocrystalline BaF_2_ exhibited a significant ISE and non-ISE region after indentation. The hardness ($H_{OP}$) and elastic modulus (E) of each plane decrease as the loading depth increases, particularly for indentation depths below 200 nm, indicating a pronounced ISE. Furthermore, the loading depth had minimal effect on the elastic modulus during elastic and elastic-plastic deformation regions.
3. The anisotropic ISE of monocrystalline BaF_2_ was effectively described by Meyer’s law, the Nix-Gao model, the PSR model, and the mPSR model. However, Meyer’s law could not accurately predict the true hardness of monocrystalline BaF_2_. Nanoindentation experiments showed better agreement with the Nix-Gao model and the PSR model, which effectively described the ISE of BaF_2_. The characteristic lengths $h^{*}$ and corresponding hardness values $H_{0}$ for the (100), (110), and (111) planes were determined to be 300.4 nm and 1.1529 ± 0.45 GPa, 381.4 nm, and 0.965 ± 0.27 GPa, and 185.8 nm and 1.1783 ± 0.32 GPa, respectively. When the indentation depth did not exceed $h^{*}$, the monocrystalline BaF_2_ exhibited a significant ISE phenomenon, which weakened as the indentation depth exceeded $h^{*}$, in accordance with the Nix-Gao model.
4. The depth-independent hardness determined using the mPSR model and the predicted $H_{0}$ values from the Nix-Gao model show adequate agreement and the mPSR model has the best correlation coefficient ($R^{2}>0.9999$). Higher $H_{OP}$ values are attributed to the creation of pile-ups at comparatively higher indentation depths. Moreover, for BaF_2_ (100), BaF_2,_ (110), and BaF_2_ (111), the size impact indexes m are 0.198, 0.211, and 0.228, respectively. BaF_2_ (100) exhibits the highest hardness and size effect index m, indicating the lowest plasticity compared to BaF_2_ (110) and BaF_2_ (111).

## Figures and Tables

**Table 1:** Values of $H_{0}$ and $h^{*}$ evaluated using the Nix–Gao model and obtained from the linear fit (Figure 9).

Sample	$H_{0}\;(GPa)$	$h^{*}\;(\mu m)$	$R^{2}$
(100)	1.1529 ± 0.45	0.3004	0.945
(110)	0.965 ± 0.27	0.3814	0.9748
(111)	1.1783 ± 0.32	0.1858	0.9336

**Table 2:** Best-fit results of the parameters to Equations (15)–(17) for monocrystalline BaF_2_.

Sample	$a_{1}\;(mN/nm)$	$a_{2}\;(mN/{nm}^{2})$	${\left(H_{0}\right)}_{1}\;(GPa)$	${\left(H_{0}\right)}_{2}\;(GPa)$	$R^{2}$
(100)	$7.71\times 10^{-4}$	$2.37\times 10^{-5}$	0.899 ± 0.15	0.967	0.99941
(110)	$1.9\times 10^{-3}$	$2.18\times 10^{-5}$	0.816 ± 0.16	0.890	0.99998
(111)	$1.29\times 10^{-3}$	$2.25\times 10^{-5}$	0.853 ± 0.13	0.918	0.99993

**Table 3:** Best-fit results of the parameters to Equations (18)–(20) for monocrystalline BaF_2_.

Sample	$a_{0}\;(mN)$	$a_{1}\;(mN/nm)$	$a_{2}\;(mN/{nm}^{2})$	${\left(H_{0}\right)}_{1}\;(GPa)$	${\left(H_{0}\right)}_{2}\;(GPa)$	$R^{2}$
(100)	0.677	$-1.3\times 10^{-3}$	$2.48\times 10^{-5}$	1.01 ± 0.098	1.012	0.99995
(110)	0.191	$1.46\times 10^{-3}$	$2.24\times 10^{-5}$	0.915 ± 0.092	0.914	0.99999
(111)	0.052	$1.84\times 10^{-3}$	$2.27\times 10^{-5}$	0.928 ± 0.094	0.927	1

## Data Availability

The authors confirm that the data supporting the findings of this study are available within the article. All data reported in this study are available upon request by contact with the first author.
